# Supplementary material for: The Chimpanzee SIV Envelope Trimer: Structure and Deployment as an HIV Vaccine Template
Source: Cell Rep. 2019 May 21;27(8):2426–2441.e6. doi: 10.1016/j.celrep.2019.04.082 (PMC6533203; doi:10.1016/j.celrep.2019.04.082)
Supplement: Document S1. Figures S1–S9 and Tables S1 and S2 [file mmc1.pdf]

Cell Reports, Volume *27*

Supplemental Information

The Chimpanzee SIV Envelope Trimer:

Structure and Deployment as an HIV Vaccine Template

Raiees Andrabi, Jesper Pallesen, Joel D. Allen, Ge Song, Jinsong Zhang, Natalia de Val, Gavin Gegg, Katelyn Porter, Ching-Yao Su, Matthias Pauthner, Amanda Newman, Hilary Bouton-Verville, Fernando Garces, Ian A. Wilson, Max Crispin, Beatrice H. Hahn, Barton F. Haynes, Laurent Verkoczy, Andrew B. Ward, and Dennis R. Burton

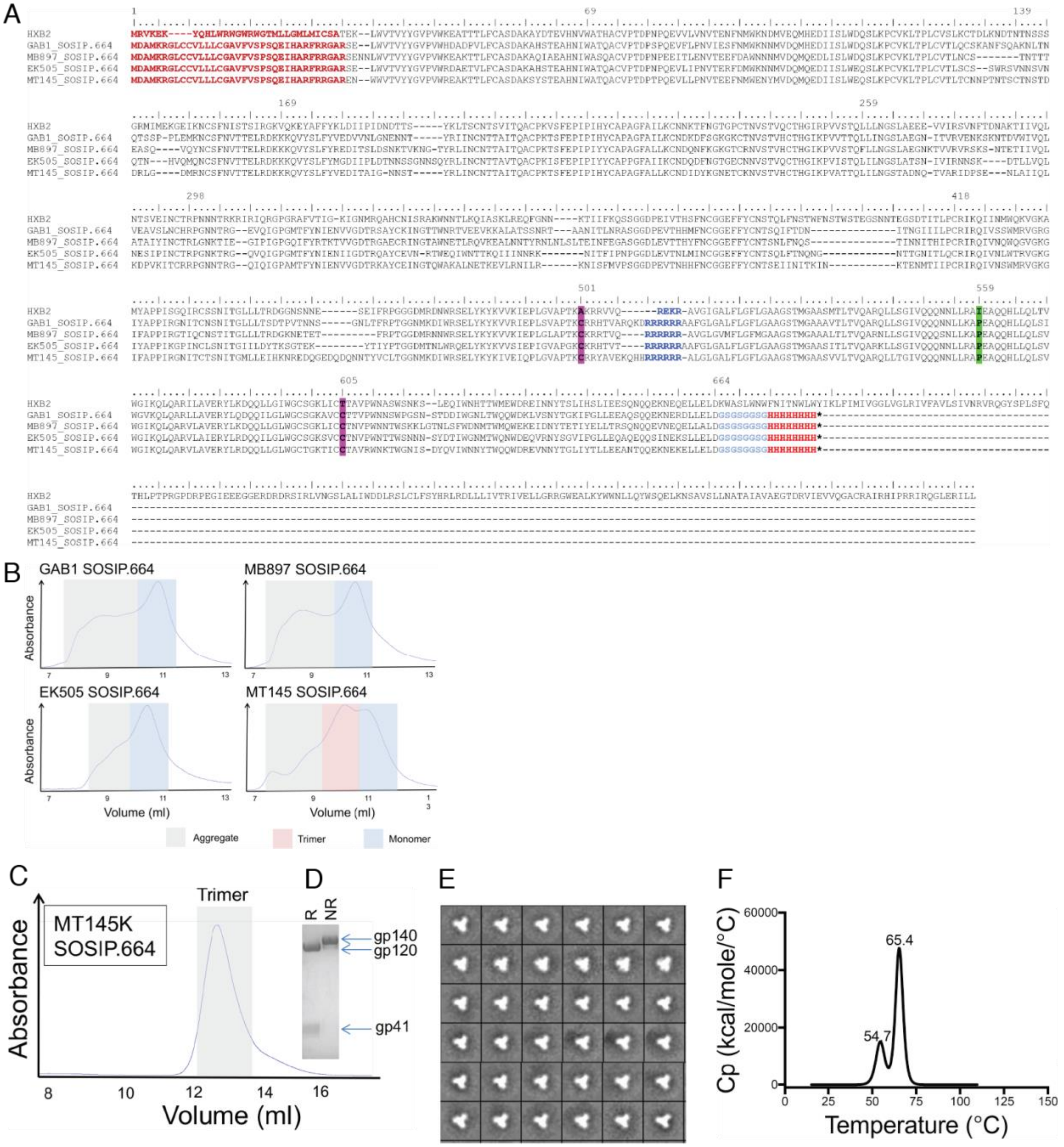

Figure S1. Design and purification of SIVcpz*Ptt* envelope derived SOSIP.664 trimers and characterization of MT145K trimer. Related to Figures 1 and 2.

A. Amino-acid sequence alignment of 4 SIVcpz*Ptt* envelope sequences (GAB1, MB897, EK505 and MT145) with reference HIV Env sequence, HXB2, showing SOSIP.664 trimer stabilizing modifications. The soluble SOSIP.664 trimer modifications include: (i) incorporation of a disulfide bond between residue 501 of gp120 (A501C) and residue 605 of gp41 (T605C), (ii) replacing naturally occurring gp120 and gp41 cleavage site with R6-cleavage site (shown in blue), (iii) I559P substitution in the gp41, (iv) and a truncation in the gp41 subunit at residue 664.

B. Size Exclusion Chromatography (SEC) profiles of *Gallanthus nivalis* lectin (GNL) purified trimers on Superdex 200 Increase 10/300 GL column. The SEC profiles show the aggregate/trimer-dimer, trimer and monomer peaks of the GNL-purified proteins. The MT145 SOSIP.664 trimer showed substantial protein fractions that assemble as a trimer.

C. Size exclusion chromatography (SEC) of PGT145 antibody purified MT145K SOSIP.664 trimer reveals trimer eluting as a single peak with no aggregation.
D. SDS-PAGE of MT145K trimer protein under non-reducing (NR) and reducing (R) conditions (reducing agent DTT was added). The MT145K trimer is efficiently cleaved into gp120 and gp41 subunits.
E. Negative Stain Electron-Microscopy (NS-EM) of MT145K trimer: 2D class averages show that trimers adopt well-ordered, native-like conformations.
F. Thermostablity of the MT145K trimer. Thermal denaturation of the MT145K trimer by Differential Scanning Calorimetry (DSC) reveals a major trimer population melting at a T*m* of 65.4 °C.

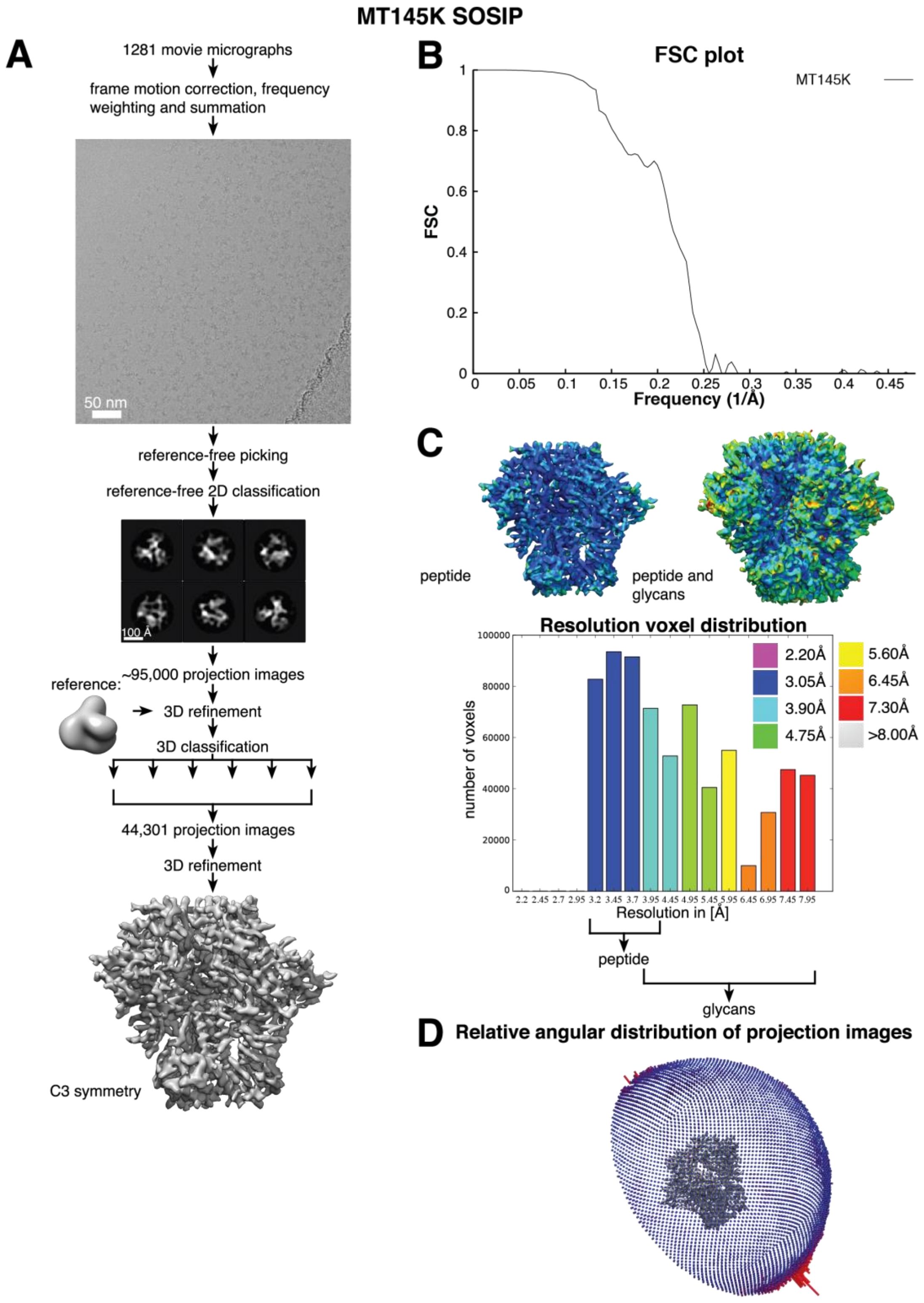

Figure S2. Structural analysis of MT145K by cryo-electron microscopy. Related to Figure 2.
A. Cryo-EM data processing flow diagram resulting in a density map at ~4.1Å global resolution.
B. FSC between two independently refined data half sets.
C. Local resolution in the MT145K density map. The peptide part of MT145K is resolved primarily in the 3.0-4.0 Å range, whereas the glycan shield overall is resolved to significantly lower resolutions.
D. Angular distribution of the angularly refined data class giving rise to the final reconstruction.

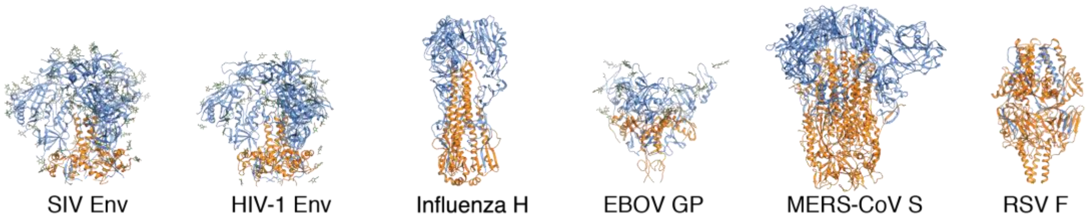

Figure S3. Comparison of Env trimer architectures of class I fusion proteins. Related to Figure 2.
Prefusion SIV Env (MT145K; current study) follows an overall organization similar to other known prefusion class I fusion proteins. Fusion subunits (shown in orange) are most commonly organized membrane-proximally (RSV F deviating somewhat from this trend). Fusion subunits are largely shielded and capped by receptor-recognizing subunits (shown in cornflower blue) in the meta-stable prefusion state. SIV Env is most similar to HIV Env; however, MT145K is less compact than the displayed HIV Env trimer (BG505 isolate). The more open organization of MT145K may be the result of its fusion peptide being inserted into a pocket at the gp120/gp41 interface as opposed to the fusion peptide found on the outside of the HIV Env trimer (MT145K: current study, HIV: PDB 4ZMJ (Kwon et al., 2015); Influenza A H1N1: PDB 1RD8 (Stevens et al., 2004); Ebola virus GP: PDB 5KEL (Pallesen et al., 2016); MERS-CoV S: PDB 5W9J (Pallesen et al., 2017); RSV F: PDB 4JHW (McLellan et al., 2013).

A

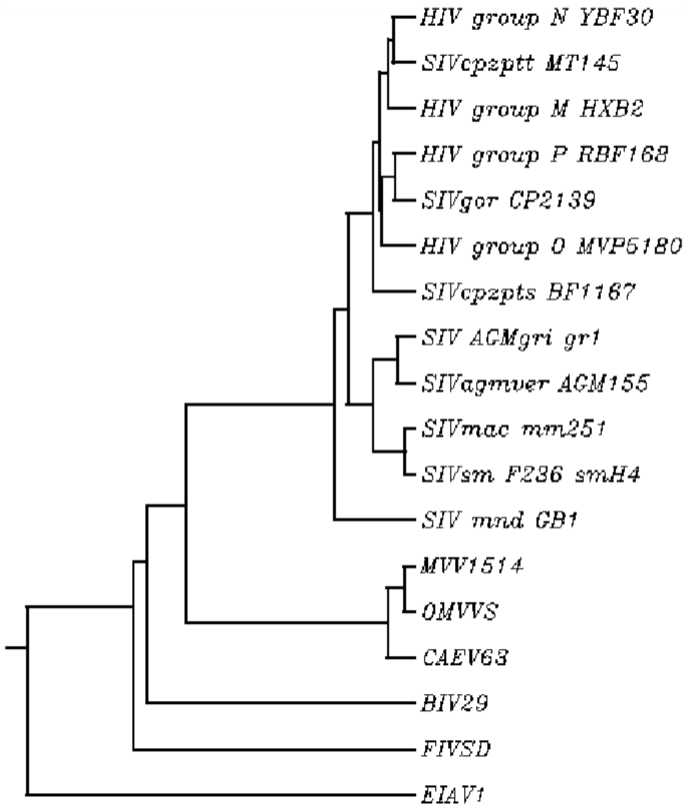

B

Env length (AA)	PNGS	Glycan density
842	29	3.44
855	27	3.16
856	31	3.62
882	30	3.40
872	30	3.44
876	32	3.65
884	26	2.94
854	24	2.81
768	22	2.86
881	24	2.72
885	24	2.71
821	20	2.44
982	29	2.95
990	28	2.83
942	28	2.97
904	19	2.10
854	22	2.58
859	19	2.21

Figure S4. Phylogeny of lentiviruses and the glycan shield density. **Related to Figure 3.**

A. Phylogenetic relationships among the envelope sequences derived from various lentiviruses; tree constructed by maximum likelihood method. These lentiviruses infect host species that include equine (EIAV), feline (FIV), bovine (BIV), caprine/ovine (CAEV, OMVVS and MVV), simian (SIV) and humans (HIV).

B. The number of amino acids and the number of Potential N-linked Glycan Sites (PNGS: predicted by N-GlycoSite tool at Los Alamos HIV database) in the full-length envelopes are listed for each lentivirus. The glycan shield density for each virus is represented as % Env residues that encode PNGS motifs and was calculated by (number of PNGS / Env length x 100). The glycan shield density shows a gradual increase from EIAV through CAEV, and plateaus in some of the SIV species including SIVcpz*Ptt*, the one that has been shown to have crossed into humans.

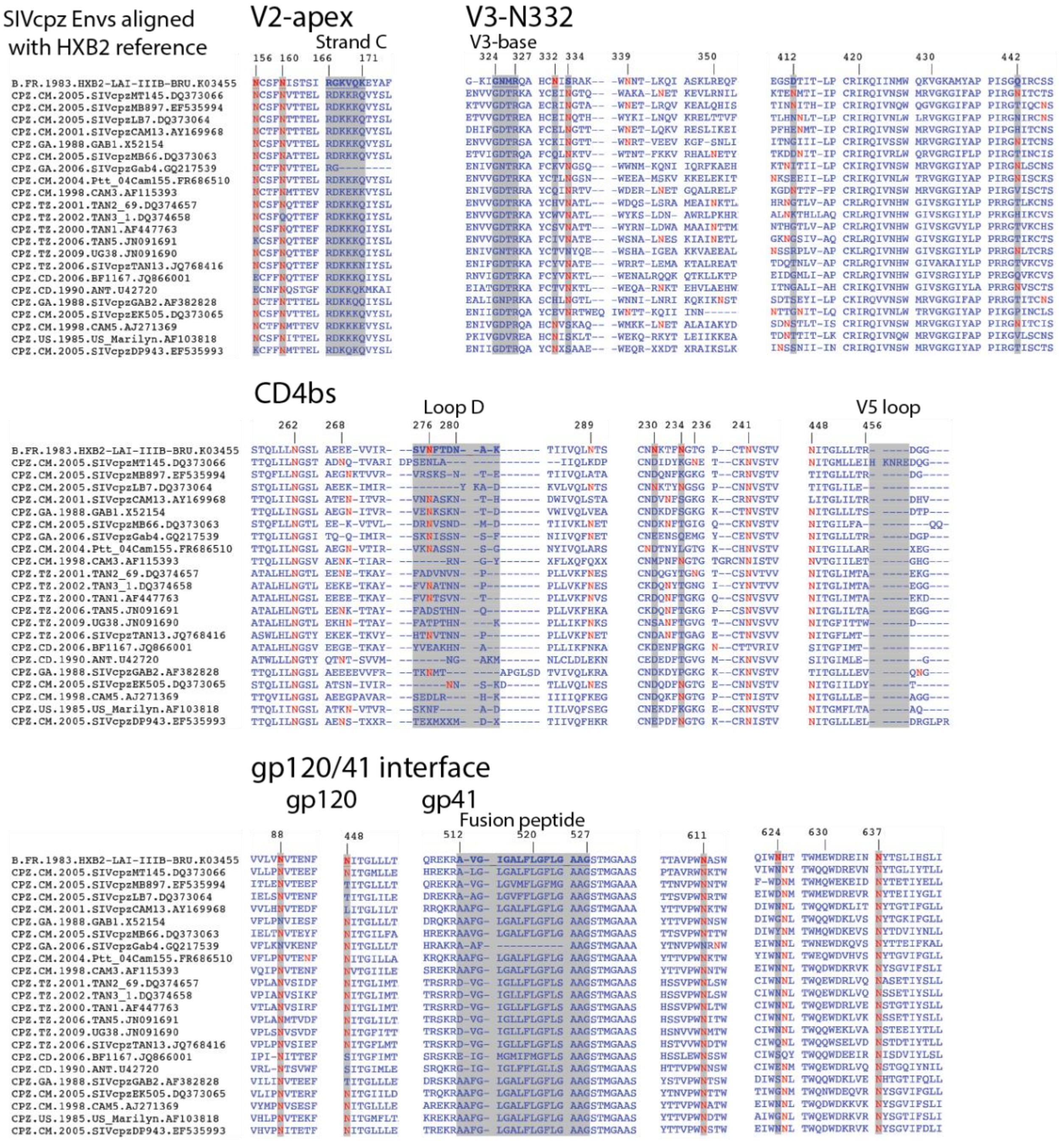

Figure S5. Amino-acid sequence alignment of SIV envelopes with HXB2 HIV Env as a reference depicting SIV Env residues equivalent to HIV bnAb epitopes. Related to Figures 3, 4 and 5.

The amino-acid alignment of SIVcpz*Ptt* envelope sequences compared to the HXB2 reference sequence. The protein residues (highlighted in grey) and the N-glycosylation sites (depicted in red) for each bnAb epitope region (V2-apex, V3-N332, CD4bs and gp120-gp41 interface) are shown.

		MT145K			BG505
		Virus	Trimer	gp120	Trimer
Epitope	Antibody	IC50	EC50	EC50	IC50
V2 Apex	PG9	0.008	0.055	0.792	0.03
	PG16	0.005	0.054	>10	0.01
	CH01	0.173	0.187	>10	0.53
	CH02	0.271	0.487	>10	0.46
	CH03	0.165	0.714	>10	>10
	CH04	0.256	0.453	>10	0.50
	PGT141	5.558	0.083	>10	0.04
	PGT142	5.779	0.075	>10	0.05
	PGT143	5.837	0.046	>10	0.04
	PGT144	>10	>10	>10	1.78
	PGT145	0.151	0.029	>10	0.02
	PGDM1400	0.010	0.018	>10	<0.003
	CAP256.01	0.121	0.130	>10	>10
	CAP256.02	0.013	0.049	>10	>10
	CAP256.03	<0.003	0.044	>10	3.25
	CAP256.04	0.004	0.076	>10	5.49
	CAP256.05	0.004	0.036	>10	>10
	CAP256.06	1.624	0.070	>10	>10
	CAP256.07	0.023	0.099	>10	>10
	CAP256.08	<0.003	0.037	>10	0.01
	CAP256.09	<0.003	0.041	>10	0.07
	CAP256.10	0.016	0.062	>10	>10
	CAP256.11	0.026	0.053	>10	>10
	CAP256.12	0.440	0.090	>10	>10
	CAP256.I1	3.713	0.122	>10	>10
	CAP256.I2	0.026	0.056	>10	>10
	2909	>10	>10	>10	>10
	C108G	>10	>10	>10	>10
	830A	>10	>10	>10	>10
	697	>10	>10	>10	>10

		MT145K			BG505
		Virus	Trimer	gp120	Trimer
Epitope	Antibody	IC50	EC50	EC50	IC50
V3-N332	PGT121	>10	>10	>10	0.03
	PGT122	>10	>10	>10	0.05
	PGT123	>10	>10	>10	4.74
	PGT124	>10	>10	>10	>10
	PGT125	>10	>10	>10	0.09
	PGT126	>10	>10	>10	0.45
	PGT127	>10	>10	>10	0.09
	PGT128	>10	>10	>10	0.05
	PGT130	>10	>10	>10	0.09
	PGT131	>10	>10	>10	>10
	PGT133	>10	>10	>10	0.01
	10-1074	>10	>10	>10	0.03
	PGT135	>10	>10	>10	>10
	PGT136	>10	>10	>10	>10
	PGT137	>10	>10	>10	>10
	2G12	>10	>10	>10	>10
V3	447-52D	>10	>10	>10	>10
	2557	>10	>10	0.483	>10
	4022	>10	>10	>10	>10
	3074	>10	>10	0.451	>10
	3791	>10	>10	>10	>10
	3904	>10	>10	0.242	>10
	4508	>10	>10	>10	>10
	537	>10	>10	>10	>10
	15e	>10	>10	>10	>10
	19b	ND	>10	>10	>10
	14e	ND	>10	0.138	>10

		MT145K			BG505
		Virus	Trimer	gp120	Trimer
Epitope	Antibody	IC50	EC50	EC50	IC50
CD4bs	VRC01	>10	>10	>10	0.07
	VRC03	>10	>10	>10	1.82
	VRC06	>10	>10	>10	>10
	HJ16	>10	>10	>10	>10
	12A12	>10	>10	>10	0.04
	NIH45-46	>10	>10	>10	0.02
	PGV04	>10	>10	>10	0.05
	CH103	>10	>10	>10	3.72
	3BNC60	>10	>10	>10	0.01
	3BNC117	>10	>10	>10	0.01
CD4bs	b6	>10	>10	>10	>10
	b12	>10	>10	>10	>10
	F105	>10	>10	>10	>10
CD4i	A32	>10	>10	>10	>10
	17b	>10	8.834	5.8	>10
	E51	>10	>10	>10	>10
CD4bs	CD4-IgG2	0.456	0.562	6.500	ND

		MT145K			BG505
		Virus	Trimer	gp120	Trimer
Epitope	Antibody	IC50	EC50	EC50	IC50
Gp120-41	PGT151	>10	1.427	>10	<0.003
	PGT152	>10	>10	>10	<0.003
	PGT153	>10	>10	>10	0.028
	PGT154	>10	>10	>10	<0.003
	PGT155	>10	>10	>10	<0.003
	PGT156	>10	>10	>10	<0.003
	PGT157	>10	>10	>10	<0.003
	PGT158	>10	>10	>10	<0.003
	8ANC195	>10	>10	>10	0.091
	35022	<0.003	0.337	>10	>10
Gp41	F240	>10	>10	>10	>10
Dengue	DEN3	>10	>10	>10	>10

IC50/EC50	Color Scale
>10	
10	
1	
0.1	
0.01	
0.003	
<0.003	
ND	Not determined

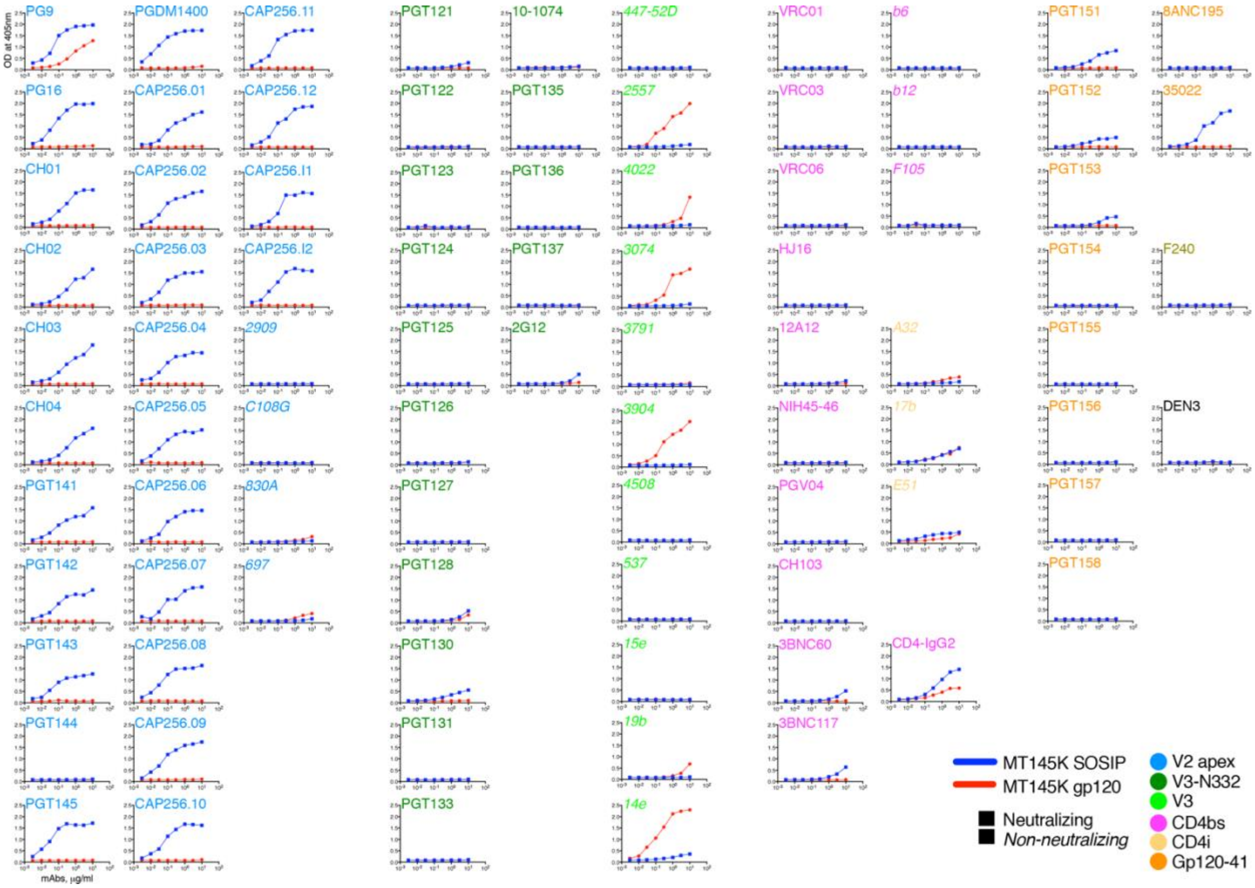

Figure S6. Antigenic profile of various MT145K Env forms with HIV Env-specific mAbs. Related to Figure 4. Neutralizing and non-neutralizing HIV Env-specific mAbs targeting various epitope specificities, including V2-apex, V3-N332, linear V3, CD4bs, CD4i and gp120-gp41 interface were tested with MT145K and BG505 Env-encoding pseudoviruses in a neutralization assay and against the MT145K SOSIP trimer and MT145K gp120 monomer by ELISA. The reciprocal IC_{50} neutralization titers against the MT145K and BG505 viruses and the 50% ELISA binding titers (EC_{50} binding with trimer and gp120 proteins) for each mAb are shown. ELISA binding curves of the HIV Env specific mAbs to the soluble MT145K trimer (blue) and its monomeric gp120 (red) protein. HIV Env mAbs specificities are shown in different colors. V2-apex directed mAbs display a strong binding with the MT145K trimer. Binding curves also reveal weak but detectable binding activities of the mAbs recognizing various HIV Env specificities (PGT121, PGT128, PGT130, 2G12, 14e, 3BNC60, 3BNC117, E51, PGT152 and PGT153).

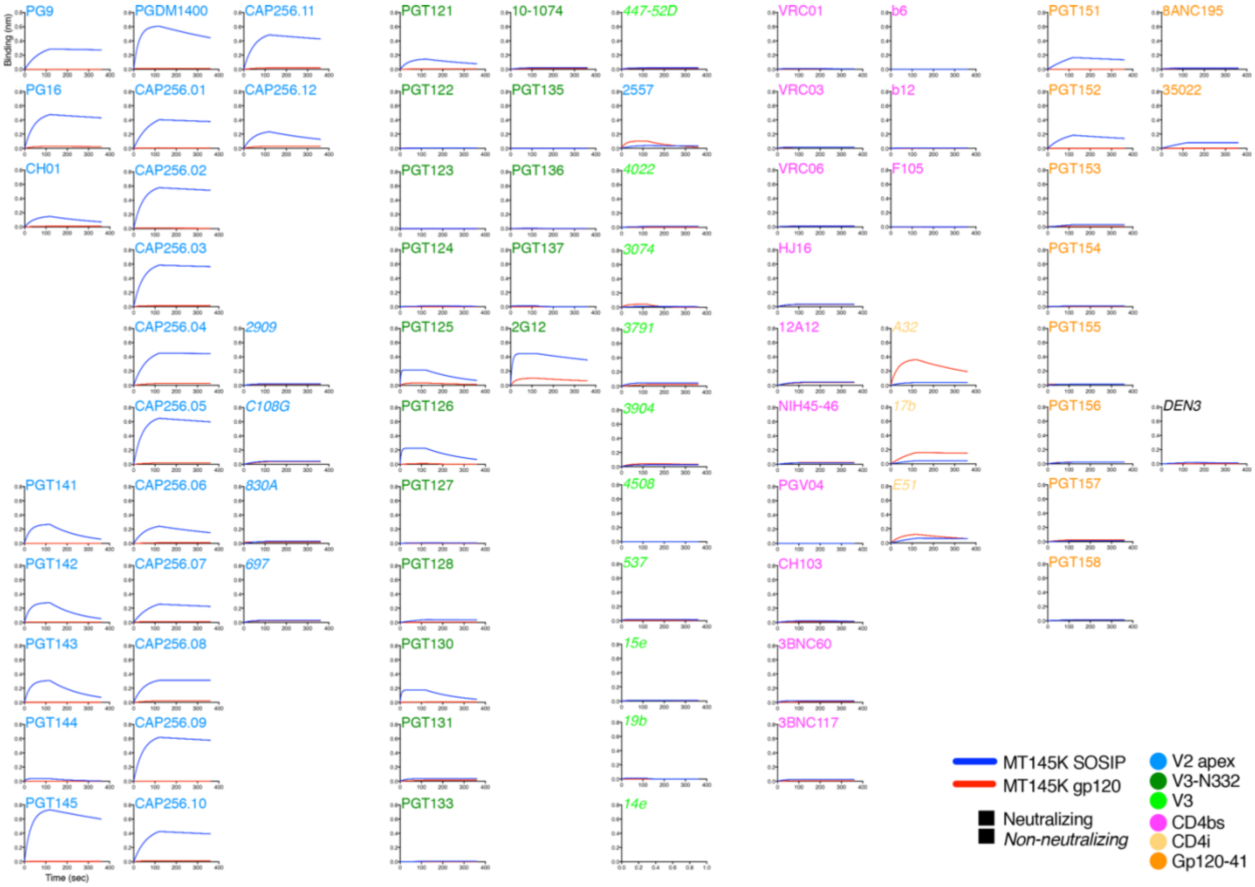

Figure S7. BioLayer Interferometry (BLI) binding responses of the HIV Env specific mAbs to the soluble MT145K trimer and MT145K monomeric gp120 proteins. Related to Figure 4.

BLI or octet binding curves (association: 120s; (0-120) and dissociation: 240s; (120-360)) of the HIV Env specific mAbs with soluble MT145K trimer (blue) and its monomeric gp120 (red) protein. 10μg/ml final concentration of the HIV mAbs recognizing a range of Env epitope specificities (mAbs names shown in different colors indicate the corresponding specificity) were captured onto an anti-human IgG-Fc sensor (AHC: ForteBio.) to achieve at least 1 RU binding response. The immobilized IgG biosensors were immersed in the 200nM MT145K trimer or 500nM MT145K gp120 solution as the analyte and the binding curves are shown as association and dissociation of Ab-protein interactions. In addition to the V2-apex directed mAbs, a few of the V3-N332 glycan mAbs, PGT121, PGT125, PGT130 and 2G12 exhibited weak binding with the MT145K trimer.

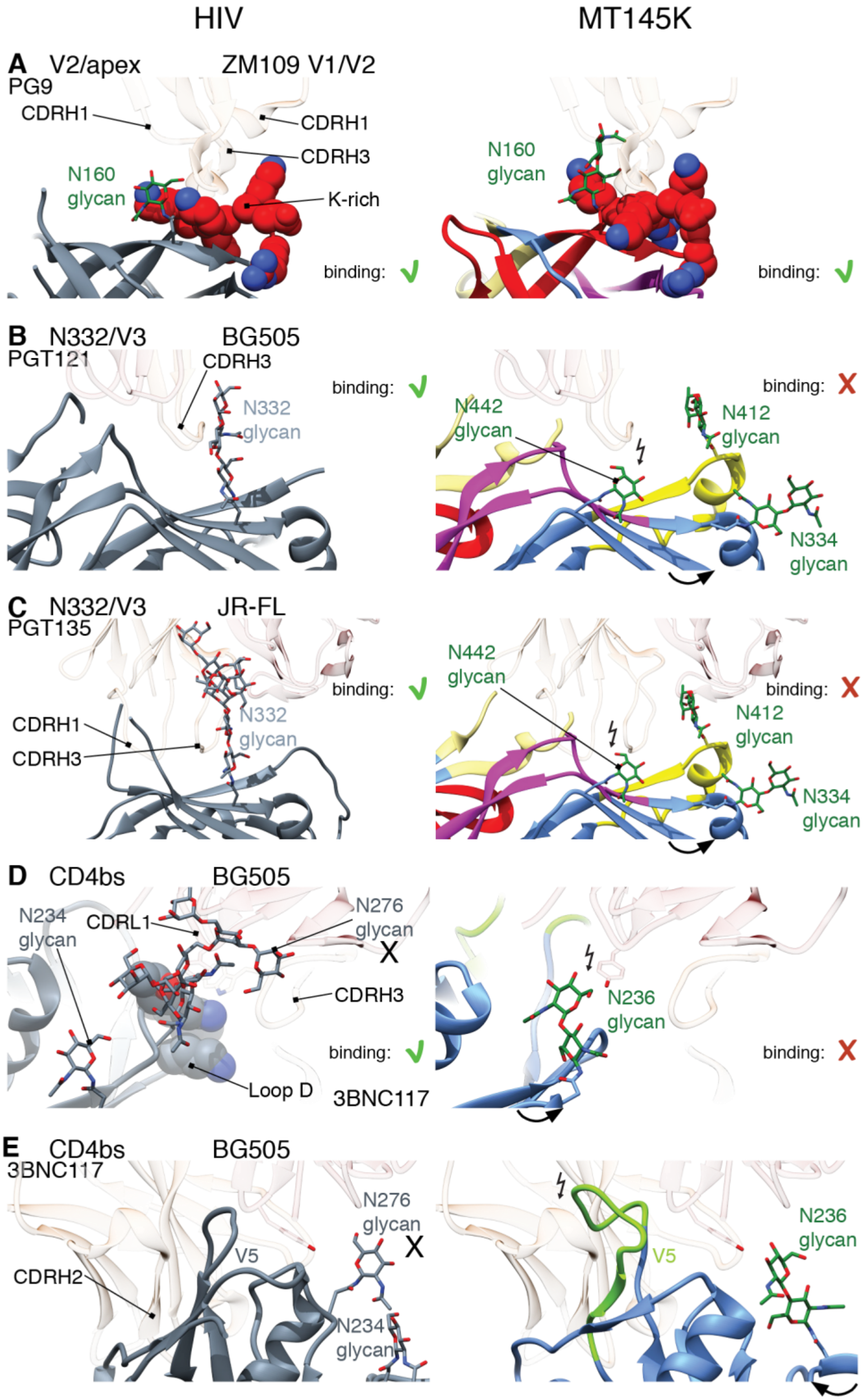

Figure S8. Molecular details of the regions on the MT145K trimer that correspond to bnAb epitopes on HIV trimers. Related to Figure 5.

A. Crystal structure of PG9 (PDB: 3U2S (McLellan et al., 2011)) bound to ZM109 V1V2-apex and docked onto BG505. The long CDRH3 of PG9 interacts with the K-rich region of the V2-apex and the glycans at N160. Both

glycan/peptide epitope elements are conserved between MT145K and HIV BG505 Env and, hence, PG9 binds strongly to both.

B-C. Crystal structures of V3-N332 bnAbs PGT121 (5T3Z (Gristick et al., 2016)) and PGT135 (PDB: 4JM2 (Kong et al., 2013)) interacting with their epitopes at the base of the V3 loop. The N332 glycan required by these bnAbs is absent on MT145K Env and the N334 glycan is projecting away from the epitopes and is not a functional substitute. In addition, the glycans N412 and N442, the latter being unique to the SIV Env, would clash with the CDRs of these bnAbs, thus preventing interaction.

D-E. Cryo-EM reconstruction of CD4bs bnAb 3BNC117, bound to BG505 trimer (PDB: 5V8M (Lee et al., 2017)). The lack of 3BNC117 binding with MT145K SIV Env trimer correlates with significant sequence variation in loop D and a clash of bnAb CDR loops with the N236 glycan that is unique to SIV. Moreover, a longer MT145 V5 loop would introduce a clash with 3BNC117 CDRH2.

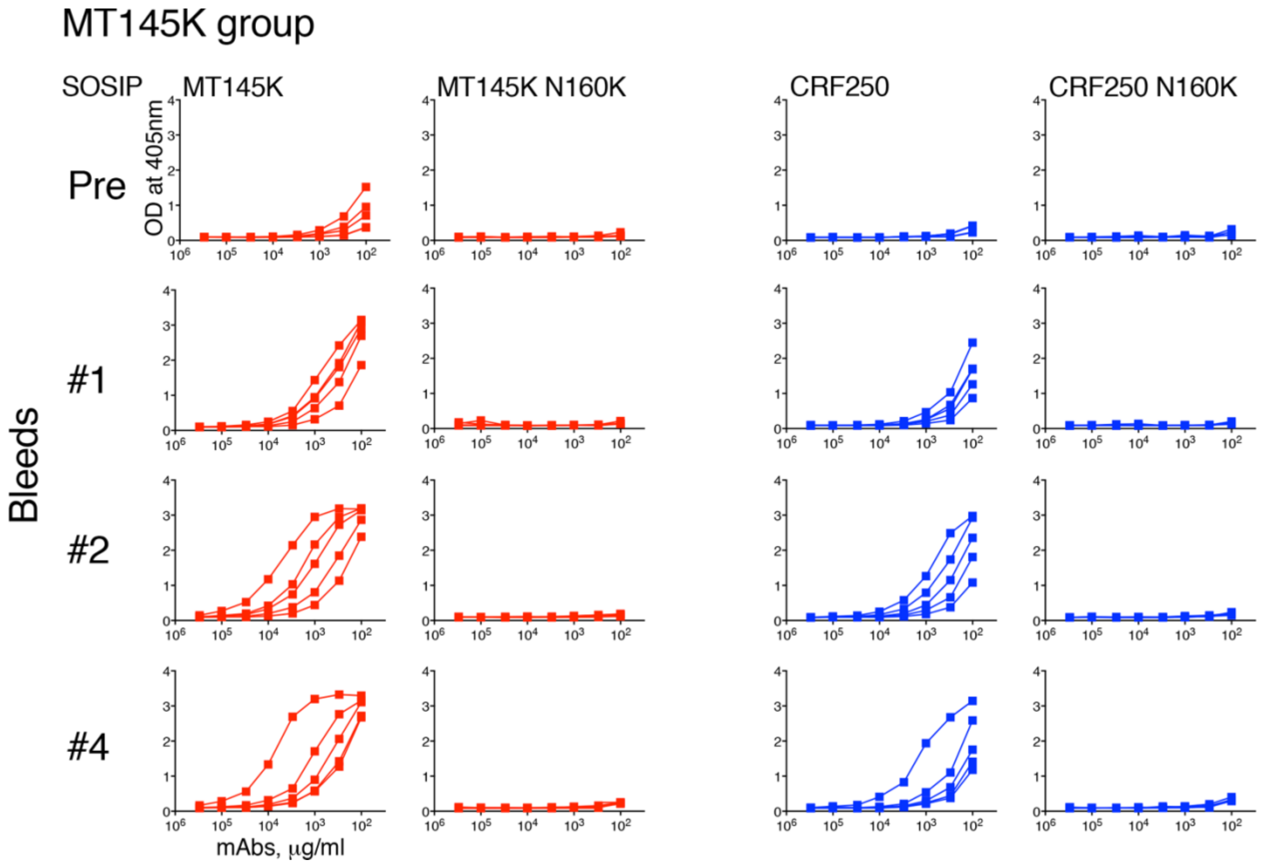

Figure S9. ELISA binding of the trimer immunized serum immune responses. Related to Figure 6.
ELISA binding curves of the MT145K trimer and HIV Env cocktail trimer-immunized CH01 UCA "HC-only" KI mice serum samples (pre-bleed (Pre), two-weeks post prime (Bleed #1), two-weeks post boost-1 (Bleed #2) and two-weeks post boost-2 (Bleed #4)) with soluble MT145K, CRF250 SOSIPs and their glycan knock-out variant (N160K) trimer proteins. Mice were immunized twice (Prime: week-0 and Boost-1: week-4) with MT145K trimer followed by boosting (boost-2 at week-8) with a 3 HIV Env-derived trimer cocktail (C108, WITO and ZM197-ZM233V1V2). Serum antibody responses mapped to the N160 glycan (part of the V2-apex bnAb core epitope) at all the immunization steps.

Map	MT145K
**Data collection**	
Microscope	FEI Titan Krios
Voltage (kV)	300
Detector	Gatan K2 Summit
Recording mode	Counting
Magnification (incl. post-magnification)	49,020
Movie micrograph pixelsize (Å)	1.02
Dose rate (e^-/[(camera pixel)*s])	10
Number of frames per movie micrograph	50
Frame exposure time (ms)	200
Movie micrograph exposure time (s)	10
Total dose (e^-/Å^2)	94
Defocus range (µm)	1.0-3.6
**EM data processing**	
Number of movie micrographs	1,281
Number of molecular projection images in map	44,301
Symmetry	C3
Map resolution (FSC 0.143; Å)	4.1
Map sharpening B-factor (Å^2)	-130
**Structure Building and Validation**	
Number of atoms in deposited model	15,192
gp120	3,547
gp41	964
glycans	553
MolProbity score	1.72 (88%)
Clashscore	3.55
EMRinger score	2.26
Deviations from ideal	
Bond length outliers	0 (0%)
Bond angles outliers	0 (0%)
Ramachandran plot	
Favored (%)	88.9
Allowed (%)	10.5
Outliers (%)	0.5

Table S1. Cryo-EM and model refinement statistics. Related to Figure 2.
Key statistics of cryo-EM data processing as well as model building and validation.

					MT145K group: Prime-MT145K (wk-0), Boost-1-MT145K (wk-4) and Boost-2 (wk-8) Cocktail of 3 HIV-1 trimers																								
				Ab	Pre-bleed (wk-0)					Bleed #1 (wk-2)					Bleed #2 (wk-6)					Bleed #3 (wk-8)					Bleed #4 (wk-10)				
Type	Virus/mutant	Subtype	Tier	CH01	13200	13201	13202	13300	13301	13200	13201	13202	13300	13301	13200	13201	13202	13300	13301	13200	13201	13202	13300	13301	13200	13201	13202	13300	13301
Autologous	MT145K	SIV	2	0.173	<100	<100	<100	<100	<100	<100	<100	<100	<100	<100	109	121	<100	135	576	<100	<100	<100	<100	340	<100	109	452	220	1788
	MT145K N160A	SIV	2	>10	<100	<100	<100	<100	<100	<100	<100	<100	<100	<100	<100	<100	<100	<100	<100	<100	<100	<100	<100	<100	<100	<100	<100	<100	<100
Boost 2 matched	WITO	B	2	0.019	173	162	<100	145	302	205	476	241	606	1095	313	1492	529	800	2846	270	350	320	310	650	105	309	300	196	1523
	C108	AE	2	0.028	<100	<100	<100	<100	<100	201	284	<100	<100	412	122	123	138	444	1413	<100	<100	149	190	890	113	182	254	229	2744
	ZM233	C	2	0.035	<100	<100	<100	<100	<100	<100	<100	<100	<100	<100	122	199	595	195	536	<100	<100	<100	<100	<100	<100	110	302	<100	191
CH01 sensitive	Q23_17	A1	1B	0.012	<100	<100	<100	<100	<100	116	383	165	369	427	197	635	260	1397	6536	153	477	471	562	3725	107	633	605	1398	>10000
	CRF250	AG	2	0.027	<100	<100	<100	<100	<100	<100	142	150	142	255	150	285	221	165	1077	<100	<100	130	<100	289	<100	<100	318	104	738
	CNE58	C	2	0.047	<100	<100	<100	<100	<100	<100	<100	<100	<100	<100	<100	<100	<100	<100	252	<100	<100	<100	<100	<100	<100	<100	126	<100	406
	BG505	A	2	0.517	<100	<100	<100	<100	<100	<100	<100	<100	<100	<100	<100	<100	<100	<100	<100	<100	<100	<100	<100	<100	<100	<100	270	<100	108
Global panel	CE703010217_B6	C	2 or 3	0.160	<100	<100	<100	<100	<100	<100	<100	<100	<100	<100	<100	113	109	<100	462	<100	<100	<100	<100	<100	<100	<100	209	115	802
	X2278_C2_B6	B	2	0.049	<100	<100	<100	<100	<100	<100	<100	<100	<100	112	200	147	342	<100	199	<100	<100	<100	<100	<100	<100	<100	151	<100	107
	CE1176_A3	C	2	0.072	<100	<100	<100	<100	<100	<100	<100	<100	<100	127	<100	<100	167	<100	128	<100	<100	<100	<100	<100	<100	<100	115	<100	<100
	398_F1_F6_20	A1	1	0.316	<100	<100	<100	<100	<100	<100	<100	<100	<100	<100	<100	<100	<100	<100	<100	<100	<100	<100	<100	<100	<100	<100	154	<100	<100
	246_F3_C10_2	A1C	2	0.981	<100	<100	<100	<100	<100	<100	<100	<100	<100	<100	<100	<100	<100	<100	<100	<100	<100	<100	<100	<100	<100	<100	109	<100	<100
	25710_2_43	C	1B or 2	1.759	<100	<100	<100	<100	<100	<100	<100	<100	<100	<100	<100	<100	<100	<100	<100	<100	<100	<100	<100	<100	<100	<100	<100	<100	<100
	CH119_10	07 BC	2	1.820	<100	<100	<100	<100	<100	<100	<100	<100	<100	<100	<100	<100	<100	<100	<100	<100	<100	<100	<100	<100	<100	<100	<100	<100	<100
	X1632_S2_B10	G	2	1.973	<100	<100	<100	<100	<100	<100	<100	<100	<100	<100	<100	<100	<100	<100	<100	<100	<100	<100	<100	<100	<100	<100	<100	<100	<100
Control	MLV4.3			>10	<100	<100	<100	<100	<100	<100	<100	<100	<100	<100	<100	<100	<100	<100	<100	<100	<100	<100	<100	<100	<100	<100	<100	<100	<100
	MT145-WT	SIV	2	ND	<100	<100	<100	<100	<100	<100	<100	<100	<100	<100	<100	<100	<100	<100	<100	<100	<100	<100	<100	<100	<100	<100	<100	<100	<100
	CRF250 N160A	AG	2	>10	<100	<100	<100	<100	<100	<100	<100	<100	<100	<100	<100	<100	<100	<100	<100	<100	<100	<100	<100	<100	<100	<100	<100	<100	<100

Table S2. Serum ID50 neutralizing antibody responses of the CH01 UCA HC-only knock-in mice immunized with the chimpanzee SIV MT145K trimer followed by HIV 3-trimer cocktail boosting. Related to Figure 7.

A group of 5 CH01 UCA HC-only knock-in mice (3 heterozygous (Het) (13200, 13201 and 13202) and 2 homozygous (Hom) (13300 and 13301) for the Ig heavy chain locus (HC)) were prime-boost immunized (prime: week-0 and boost-1: week-4), with chimpanzee MT145K trimer. The animals were further boosted (boost-2 at week-8) with an HIV Env derived 3-trimer cocktail (C108, WITO and ZM197-ZM233V1V2). The mice were immunized with 25µg of the individual trimer or HIV 3-trimer cocktail (25µg total protein) with GLA-SE as adjuvant. Neutralization of the pre- and post-immune sera were carried out against a panel of viruses in the TZM-bl reporter cell assay. The numerical values in the table represent ID_{50} neutralization titers of the pre-bleed (Pre) and post-prime (Bleed #1), post boost-1 (Bleed #2), post boost-1 prior to HIV trimer boosting (Bleed #3), and post boost-2 (Bleed #4) serum samples. Neutralization was assessed against the priming immunogen-matched autologous virus, MT145K and its N160 glycan knock-out variants, boosting immunogen-matched, CH01 Ab sensitive, and global panel HIV Env-encoding viruses. The numerical values shown in the table represent the ID_{50} serum neutralization titers and were calculated by non-linear regression method from the percent neutralizations of serum titrations with virus. The ID_{50} values of <100 indicate that 50% of neutralization was not achieved for the respective serum-virus combinations at the lowest dilution (1:100) tested in neutralization assay. The IC_{50} neutralization titers for the CH01 mature Ab against the virus panel are shown.
